# Role of TRAIL-R in Primary and Secondary Genital and Respiratory Chlamydia muridarum Infections in Mice

**DOI:** 10.1128/spectrum.01617-22

**Published:** 2022-07-25

**Authors:** Sukumar Pal, Sydni Sheff, Mufadhal Al-Kuhlani, David M. Ojcius, Luis M. de la Maza

**Affiliations:** a Department of Pathology and Laboratory Medicine, Medical Sciences I, University of California, Irvine, Irvine, California, USA; b Department of Biomedical Sciences, Arthur Dugoni School of Dentistry, University of the Pacific, San Francisco, California, USA; c Life Science Department, Fresno City College, Fresno, California, USA; University of Florida

**Keywords:** *chlamydia*, *Chlamydia muridarum*, TNF-related apoptosis inducing ligand receptor, TRAIL, genital infection, mice, respiratory infection

## Abstract

The tumor necrosis factor (TNF)-related apoptosis-inducing ligand receptor (TRAIL-R) suppresses inflammation and could therefore affect the course of Chlamydia infections and their long-term sequelae. Wild-type (WT) and *TRAIL-R*^−/−^ C57BL/6 mice were inoculated vaginally with Chlamydia muridarum; the course of the infection was followed with vaginal cultures and the presence of hydrosalpinx determined. To evaluate the role of TRAIL-R following a secondary infection, the mice were vaginally reinfected. WT and *TRAIL-R*^−/−^ male mice were also infected and reinfected in the respiratory tract, and the course of the diseases and the infections were followed. Following the primary and secondary vaginal infection, no significant differences in vaginal shedding or hydrosalpinx formation were observed between the WT and *TRAIL-R*^−/−^ mice. The WT and *TRAIL-R*^−/−^ mice mounted antibody responses in serum and vaginal washes that were not significantly different. After the primary and secondary intranasal infections of the male mice, changes in body weight were determined, and no significant differences were observed between the WT and *TRAIL-R*^−/−^ mice. Ten days after the primary and the secondary infections, the weight of the lungs and number of C. muridarum inclusion forming units (IFU) were determined. The lungs of the WT mice weighed less compared with the *TRAIL-R*^−/−^ mice following a primary infection but not after a secondary infection. No differences in the number of C. muridarum IFU in the lungs were observed between the two groups of mice. In conclusion, despite playing a role in inflammation cell-signaling pathways *in vitro*, TRAIL-R does not appear to play a major role in the susceptibility, clinical outcomes, or long-term sequelae of C. muridarum infections *in vivo*.

**IMPORTANCE** TNF-related apoptosis-inducing ligand receptor (TRAIL-R) is involved in suppressing inflammatory responses. Bacterial pathogens such as Chlamydia spp. elicit inflammatory responses in humans following genital, ocular, and respiratory infections. The inflammatory responses are important to control the spread of Chlamydia. However, in certain instances, these inflammatory responses can produce long-term sequelae, including fibrosis. Fibrosis, or scarring, in the genital tract, eye, and respiratory system results in functional deficiencies, including infertility, blindness, and chronic obstructive lung disease, respectively. The goal of this study was to determine if mice deficient in TRAIL-R infected in the genital and respiratory tracts with Chlamydia spp. suffer more or less severe infections, infertility, or lung diseases than wild-type mice. Our results show no differences between the immune responses, infection severity, and long-term sequelae between TRAIL-R knockout and wild-type animals following a genital or a respiratory infection with Chlamydia.

## INTRODUCTION

Chlamydia trachomatis is an obligate intracellular Gram-negative bacterium with a unique developmental cycle (1, 2). The elementary body (EB) is the infectious form that measures ~300 nm in diameter and is metabolically weakly active. Upon infection, the EB is internalized into an intracytoplasmic inclusion and differentiates into the reticulate body (RB), which measures around ~1,000 nm in diameter and divides by binary fission. After 8 to 10 cycles of replication, the RB converts back into the EB. Over time, the inclusion increases in size until the cells die and rupture or the chlamydial inclusion is extruded (3, 4).

More than 1.8 million cases of sexually transmitted C. trachomatis infections were reported to the Centers for Disease Control and Prevention in 2018, and the number of cases continues to increase (5). Worldwide, it is estimated that 131 million new genital infections occur every year (5, 6). Over 80% of C. trachomatis genital infections in females and ~50% of the infections in males are asymptomatic (5, 7, 8). In some patients, C. trachomatis can produce acute and chronic infections that can result in long-term sequelae, including pelvic inflammatory disease (PID), chronic abdominal pain, ectopic pregnancy, and infertility (1, 9–11). Infants born to C. trachomatis-infected women can develop conjunctivitis and pneumonia. In countries with poor sanitary conditions, C. trachomatis produces ocular infections that lead to blindness (1, 12, 13).

Factors known to affect the severity of an infection in humans, and in the mouse model, include the age of the individual, stage of the estrus cycle, bacterial load, and prior exposure to C. trachomatis (8, 14–16). In addition, data suggest that the host genetic background affects susceptibility to infection and the intensity of the immune responses (17). For example, single nucleotide polymorphisms (SNPs) in *TLR2* and *TLR9* have been found to influence the outcomes of C. trachomatis genital infections (18).

The cytokine tumor necrosis factor (TNF)-related apoptosis-inducing ligand (TRAIL) causes apoptosis by binding to TRAIL receptors (TRAIL-R) after the cytokine is secreted from normal tissue cells. It has been shown that TRAIL is involved in regulation of innate immune responses and that deficiency of TRAIL-R, in some animal models, affects infection with various pathogens and promotes susceptibility to chronic inflammation and tumorigenesis (19–23). For example, Diehl et al. (22) infected *TRAIL-R*^−/−^ mice with Listeria monocytogenes, Salmonella enterica serovar Typhimurium, Mycobacterium bovis Bacillus-Calmette-Guerin (BCG), encephalomyocarditis virus, and murine cytomegalovirus. Although they observed that *TRAIL-R*^−/−^ mice responded to certain challenges like wild-type (WT) mice, in the case of an infection with murine cytomegalovirus, *TRAIL-R*^−/−^ mice were more resistant than WT animals, as shown by a decrease in the number of viral titers in the spleen. In addition, using *TRAIL^−/−^* mice, Zheng et al. (23) investigated susceptibility to L. monocytogenes infection, based on the number of organisms in the liver and spleen and the survival time, and reported that these mice were partially resistant compared to the WT animals. Myeloid and lymphoid apoptosis, which resulted in spleen enlargement, was inhibited in the *TRAIL^−/−^* mice (23). Cardoso Alves et al. (24) found that clearance of lymphocytic choriomeningitis virus was faster, and liver pathology decreased, in *TRAIL^−/−^* mice compared with WT mice and proposed that an improvement of specific CD8^+^ T-cell responses in *TRAIL^−/−^* mice accounted for these differences. Following intranasal (i.n.) infection with Streptococcus pneumoniae, Steinwede et al. demonstrated that *TRAIL*^−/−^ mice had a decrease in lung bacterial clearance and survival in comparison with WT animals (25). Treatment of WT mice with TRAIL, or agonistic anti-DR3 monoclonal antibody (MAb) (MD5-1), significantly improved their survival following an i.n. challenge with S. pneumoniae.

We previously isolated bone marrow-derived macrophages (BMDMs) and lung fibroblasts from WT and *TRAIL-R*^−/−^ mice (26). Infection of the BMDMs and lung fibroblasts with a C. muridarum isolate, or the human pathogen C. trachomatis strain L2, led to higher levels of macrophage-inflammatory protein 2 (MIP2) mRNA expression or interleukin-1β (IL-1β) secretion from TRAIL-R-deficient cells than from WT cells. Thus, despite the effects of TRAIL-R on inflammation during infection of cells *in vitro*, here we find that infection of WT and *TRAIL-R*^−/−^ mice with C. muridarum was similar in terms of susceptibility, clinical outcomes, and long-term sequelae.

Unlike the results of chlamydial infection in mice, we had previously found that some TRAIL-R1 single nucleotide polymorphisms (SNPs) are associated with C. trachomatis infections in humans more often than other SNPs (26). This observation was supported by experiments in which TRAIL-R1 levels in human cervical epithelial cells were depleted by RNA interference. We found that TRAIL-R1 depletion in the human cells led to higher levels of IL-8 mRNA expression and protein secretion during C. trachomatis infection *in vitro* than in control human cells.

Here, to determine the role that TRAIL-R may have on a primary and secondary chlamydial infection and the long-term sequelae, we inoculated WT and *TRAIL-R*^−/−^ mice in the genital or respiratory tract with Chlamydia muridarum and followed the outcomes of the primary and secondary infections.

## RESULTS

### Susceptibility of TRAIL-R deficient mice to a C. muridarum primary vaginal infection.

To assess the susceptibility of *TRAIL-R*^−/−^ mice to a primary Chlamydia infection, four groups of 8-week-old females were inoculated intravaginally with 10^2^, 10^3^, 10^4^, or 10^5^ C. muridarum inclusion-forming units (IFU). As controls, WT C57BL/6 mice of the same age were inoculated with the same number of C. muridarum IFU. Vaginal cultures were collected, and five parameters were determined: the number of mice with positive vaginal cultures, the number of positive vaginal cultures, median days to clearance, the total number of C. muridarum IFU shed, and the number of hydrosalpinges.

**FIG 1:**
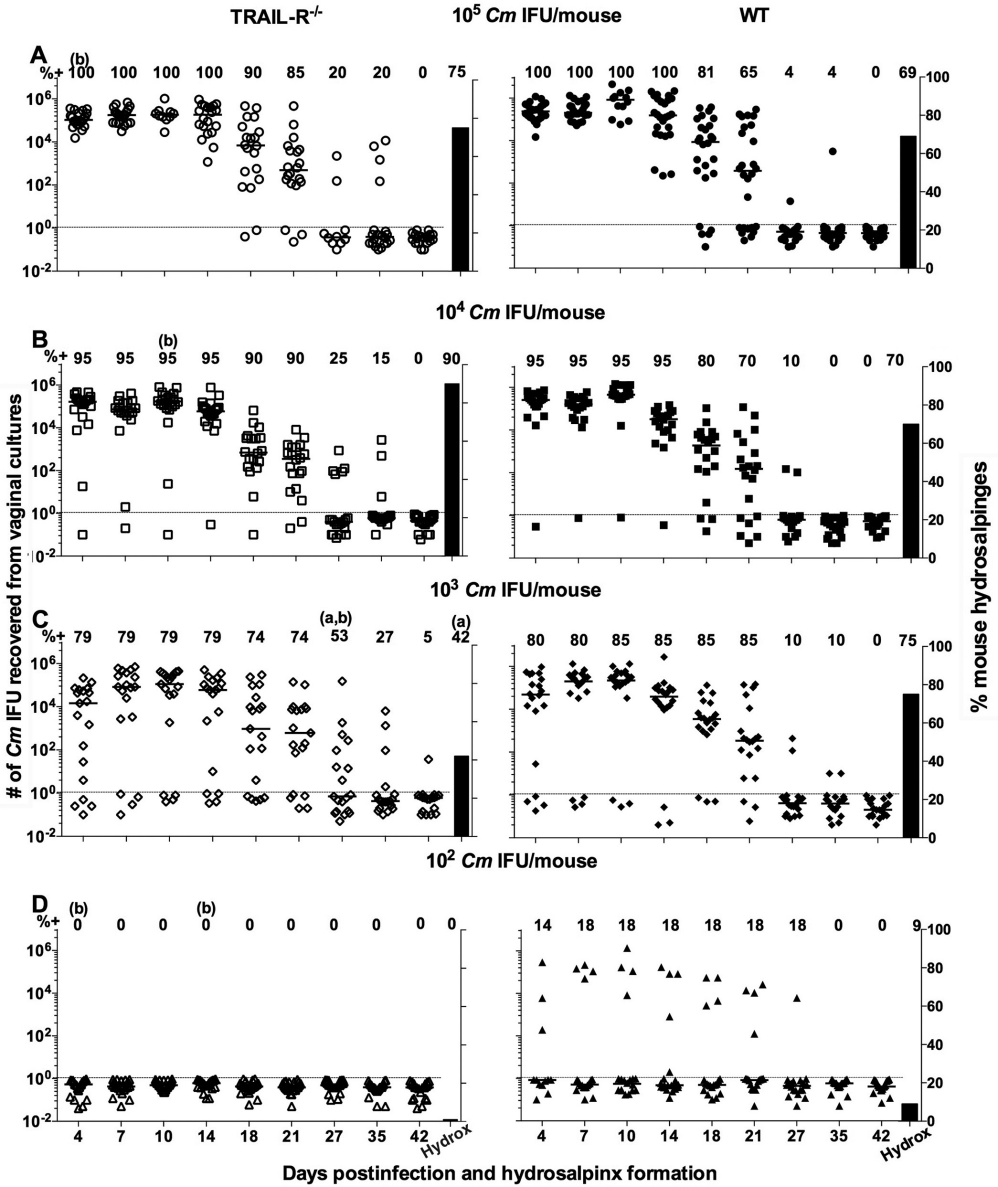
Vaginal shedding and long-term sequelae of *TRAIL-R*^−/−^ (left) and WT (right) mice following a primary vaginal infection with (A through D) 10^5^, 10^4^, 10^3^, or 10^2^ C. muridarum IFU. Vaginal cultures were collected twice a week for the first 3 weeks and then weekly for three additional weeks. The numbers along the top of each graph represent the percentages of mice with positive vaginal cultures. The horizontal lines indicate the median number of C. muridarum IFU per group. The symbols denote the number of C. muridarum IFU for each individual mouse. The dotted line indicates the limit of detection (2 C. muridarum IFU/vaginal culture). The percentage of mice with hydrosalpinx is shown as a vertical black bar. Statistically significant differences (*P* < 0.05) between WT and *TRAIL-R*^−/−^ animals are indicated as follows: ^a^, percentage of mice with positive cultures; ^b^, number of C. muridarum IFU; ^c^, percentage of mice with hydrosalpinx. Cm, C. muridarum; hydrox, hydrosalpinx.

As shown in Fig. 1 and Tables S1A and S1B in the supplemental material, no significant differences in the numbers of mice with positive vaginal cultures were observed. All mice infected with 1 × 10^5^ IFU of C. muridarum had positive vaginal cultures, and 95% (19/20) of the WT and *TRAIL-R*^−/−^ animals infected with 1 × 10^4^ IFU were positive (*P* > 0.05). Similarly, no differences were observed in the numbers of mice infected with 1 × 10^3^ IFU or with 1 × 10^2^ C. muridarum IFU.

Differences in the numbers of positive vaginal cultures between the two groups of mice were only detected in mice inoculated with 10^2^ C. muridarum IFU (Table S1B). Of the WT mice infected with 10^2^ IFU of C. muridarum, 14% (27/198) of the vaginal cultures were positive, while in the *TRAIL-R*^−/−^ mice only 1% (2/198) were positive (*P* < 0.05).

Based on the length of shedding, some differences between the WT and *TRAIL*^−/−^ mice were observed (Fig. 1 and Table S1B). In mice infected with 10^5^ IFU, the median number of days to clearance for the WT (35; range, 21 to 42) and *TRAIL-R*^−/−^ (27; range, 18 to 42) mice were different (*P* < 0.05). WT mice infected with 10^4^ C. muridarum IFU also had a longer course of shedding, with a median of 27 (range, 4 to 35) days, compared with the knockout (KO) mice (median, 27 days; range, 21 to 42 days) (*P* < 0.05). The WT and *TRAIL-R*^−/−^ mice infected with 10^2^ or 10^3^ C. muridarum IFU showed no differences in the time to clearance.

The total number of C. muridarum IFU shed over the 6-week period following infection was different between the WT and *TRAIL-R*^−/−^ mice for the groups vaginally infected with 10^4^ or 10^5^ C. muridarum IFU (Fig. 1 and Table S1B). At the two different doses, the *TRAIL-R*^−/−^ KO mice shed fewer IFU than the WT. For example, WT mice infected with 10^4^ C. muridarum IFU shed a median number of 1,223,424 IFU/mouse, while the *TRAIL-R*^−/−^ mice shed 541,581 IFU/mouse (*P* < 0.05).

At 7 weeks after infection, the mice were euthanized and their genital tracts inspected *in situ* for the presence of hydrosalpinx as a marker of upper genital tract pathology (Fig. 1 and Table S1B). For the WT and *TRAIL-R*^−/−^ mice, vaginally infected with 10^5^, 10^4^, 10^3^, or 10^2^ C. muridarum IFU, the percentages of animals that had hydrosalpinx were 69% versus 75%, 70% versus 90%, 75% versus 42%, and 9% versus 0%, respectively (*P* > 0.05).

### Effects of a secondary vaginal infection with C. muridarum in WT and *TRAIL-R^−/−^* mice.

**FIG 2:**
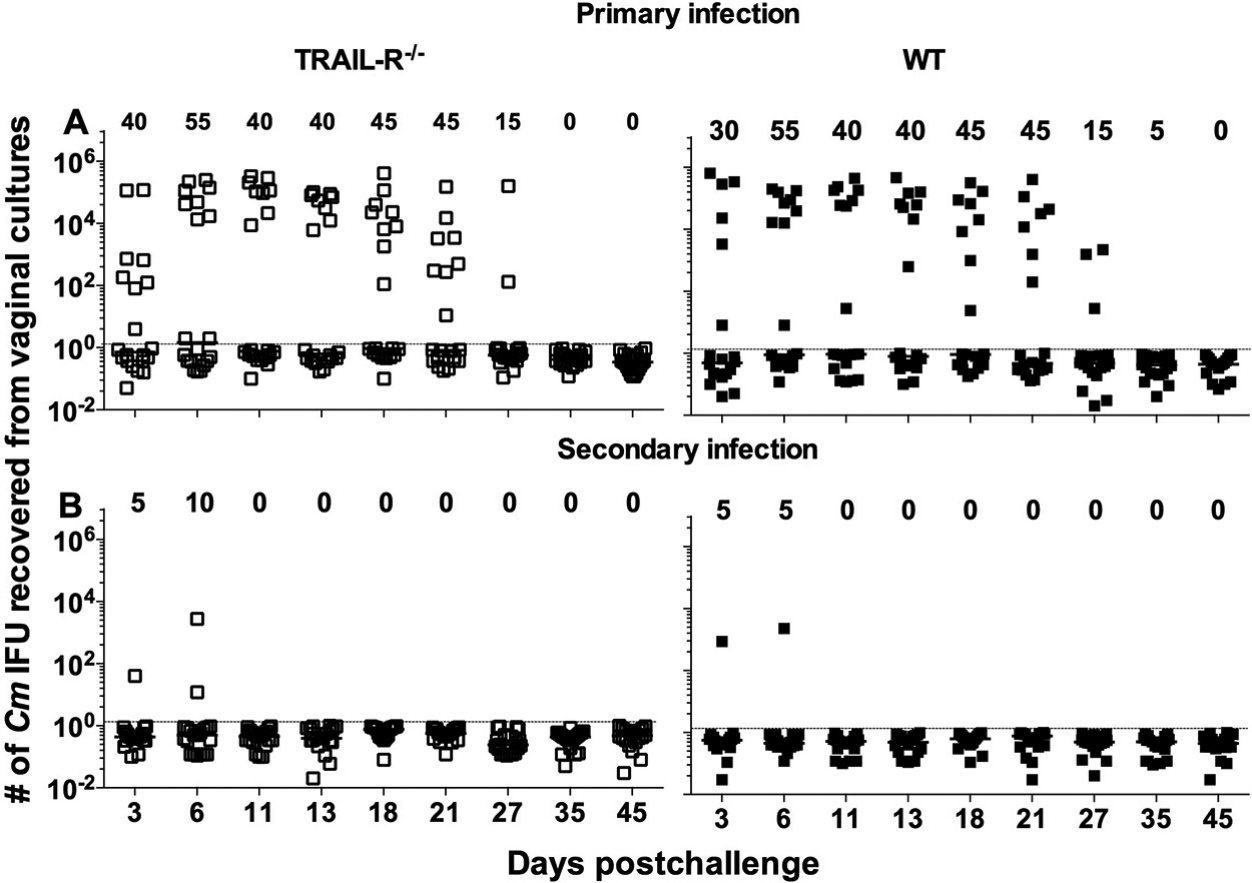
Vaginal shedding of *TRAIL-R*^−/−^ (left) and WT (right) mice following a primary (A) and a secondary (B) vaginal infection with C. muridarum. Mice were infected vaginally with 10^2^ IFU of C. muridarum, followed 6 weeks later by a secondary vaginal infection with 10^3^ IFU. The numbers at the top of each graph represent the percentages of mice with positive vaginal cultures. The horizontal lines indicate the median number of C. muridarum IFU per group. The symbols denote the number of C. muridarum IFU for each individual mouse. The dotted line indicates the limit of detection (2 IFU/vaginal culture).

To evaluate the role of TRAIL-R in adaptive immunity, WT and *TRAIL-R*^−/−^ mice were first inoculated vaginally with 2 × 10^2^ IFU of C. muridarum and then reinfected 6 weeks later with 1 × 10^3^ IFU by the same route (Fig. 2; Tables S2A and B).

Following the primary infection, 45% (9/20) of the WT mice and 65% (13/20) of the *TRAIL-R*^−/−^ mice had positive vaginal cultures (*P* > 0.05). No differences in the percentage of positive cultures were detected between the WT (27% [49/180]) and *TRAIL-R*^−/−^ mice (31% [56/180]) (*P* > 0.05). Similarly, the median time in days to clearance and the total number of C. muridarum IFU recovered were not significantly different between the two groups of mice.

Following a secondary vaginal infection, based on the number of mice with positive vaginal cultures (WT mice, 5% [1/20]; *TRAIL-R*^−/−^ mice, 10% [2/20]), no differences were observed between the two groups of animals (*P* > 0.05). The median number of days to negative vaginal cultures was the same for the WT and *TRAIL-R*^−/−^ mice (4; range, 4 to 11). In addition, no significant differences were found between the number of positive vaginal cultures in WT (1.1% [2/180]) versus *TRAIL-R*^−/−^ (1.7% [3/180]) mice or the median number of C. muridarum IFU shed per mouse (WT, <2 IFU; range, <2 to 3,161 IFU) versus *TRAIL-R*^−/−^ (<2 IFU; range, <2 to 2,818 IFU).

Based on the four parameters used to evaluate vaginal shedding, the percentage of mice with positive cultures, time to a negative culture, number of positive cultures, and number of C. muridarum IFU detected, both the WT and *TRAIL-R*^−/−^ mice were significantly protected against a secondary infection (*P* < 0.05).

To assess the long-term sequelae, the genital tracts of the mice were evaluated *in situ* following the secondary infection. The number of mice that had hydrosalpinx (WT, 25% [5/20]; *TRAIL-R*^−/−^, 35% [7/20]) was not significantly different between the two groups (*P* > 0.05).

### Antibody responses following primary and secondary vaginal infections of WT and *TRAIL-R^−/−^* mice with C. muridarum.

To determine if differences in the humoral immune responses were mounted by the WT versus *TRAIL-R*^−/−^ mice, blood was collected at 6 weeks following the vaginal primary infection with C. muridarum. IgG, IgG1, and IgG2c antibody titers were similar in WT and *TRAIL-R* KO mice following primary infection with various dosages of C. muridarum (Table S3). For example, in the WT mice infected with 10^5^ IFU of C. muridarum, the IgG geometric mean titer (GMT) was 32,254 and for the *TRAIL-R*^−/−^ mice, 40,637. Only the mice inoculated with 10^2^ IFU showed significant differences in antibody titers, reflecting the higher number of positive vaginal cultures in the WT versus the *TRAIL-R*^−/−^ mice. The *TRAIL-R^−/−^* mice inoculated with 10^2^ IFU had the following GMTs: IgG, 112; IgG1, <100; and IgG2c, <100. By contrast, the WT mice inoculated with 10^2^ IFU had significantly higher GMTs: IgG, 3,592; IgG1, 238; and IgG2c, 3,676 (*P* < 0.05). As indicated by the IgG2c/IgG1 ratios, the WT and *TRAIL-R*^−/−^ mice vaginally infected once with C. muridarum mounted robust Th1-biased humoral responses.

To determine if a primary infection affected the antibody responses of a secondary infection, mice were first inoculated vaginally with 1 × 10^2^ C. muridarum IFU and subsequently challenged with 2 × 10^3^ IFU. As shown in Table S4, no significant differences in serum IgG, IgG1, or IgG2c were observed between the WT and *TRAIL-R^−/−^* mice. The IgG2c/IgG1 ratios were also similar in both types of mice.

The IgG and IgA antibody titers were determined in pooled vaginal washes following primary and secondary vaginal infections with 2 × 10^2^ and 1 × 10^3^ C. muridarum IFU, respectively. The IgG and IgA titers in both the WT and *TRAIL-R^−/−^* mice ranged from 160 to 320 and were not significantly different (Table S5).

### Susceptibility of *TRAIL-R^−/−^* mice to a C. muridarum primary respiratory infection.

**FIG 3:**
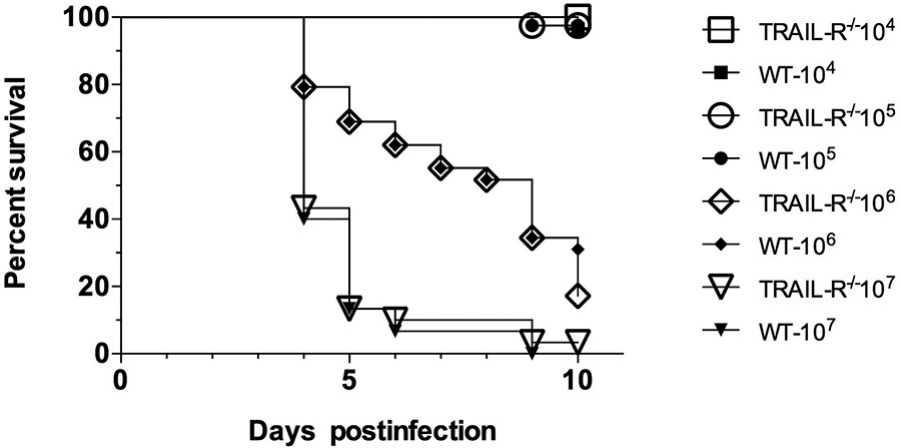
Determination of the C. muridarum LD_50_ in WT and *TRAIL-R*^−/−^ mice following an intranasal challenge. Four groups of mice were inoculated in the nostrils with 10^4^, 10^5^, 10^6^, or 10^7^ C. muridarum IFU. The animals were observed daily for 10 days and the percentage survival determined.

Since the TRAIL-R is expressed at various levels in different tissues, we also inoculated the WT and *TRAIL-R*^−/−^ mice with C. muridarum intranasally. To determine the median lethal dose (LD_50_), groups of 5 to 10 mice were inoculated i.n. with 10^4^, 10^5^, 10^6^, or 10^7^ C. muridarum IFU. As shown in Fig. 3, the survival of the WT and *TRAIL-R*^−/−^ mice was the same for all the doses tested. For the WT and *TRAIL-R*^−/−^ mice, the LD_50_ values were 1.9 × 10^5^ and 2.5 × 10^5^, respectively (*P* > 0.05).

To assess the outcome of the primary respiratory tract infection, three groups of WT and *TRAIL-R*^−/−^ mice were inoculated in the nostrils with 10^2^, 10^4^, or 10^5^ C. muridarum IFU. The course of the infection was followed for 10 days by determining changes in body weight. At 10 days postinfection (dpi), the mice were euthanized, their lungs collected and weighed, and the numbers of C. muridarum IFU in the lungs determined.

**FIG 4:**
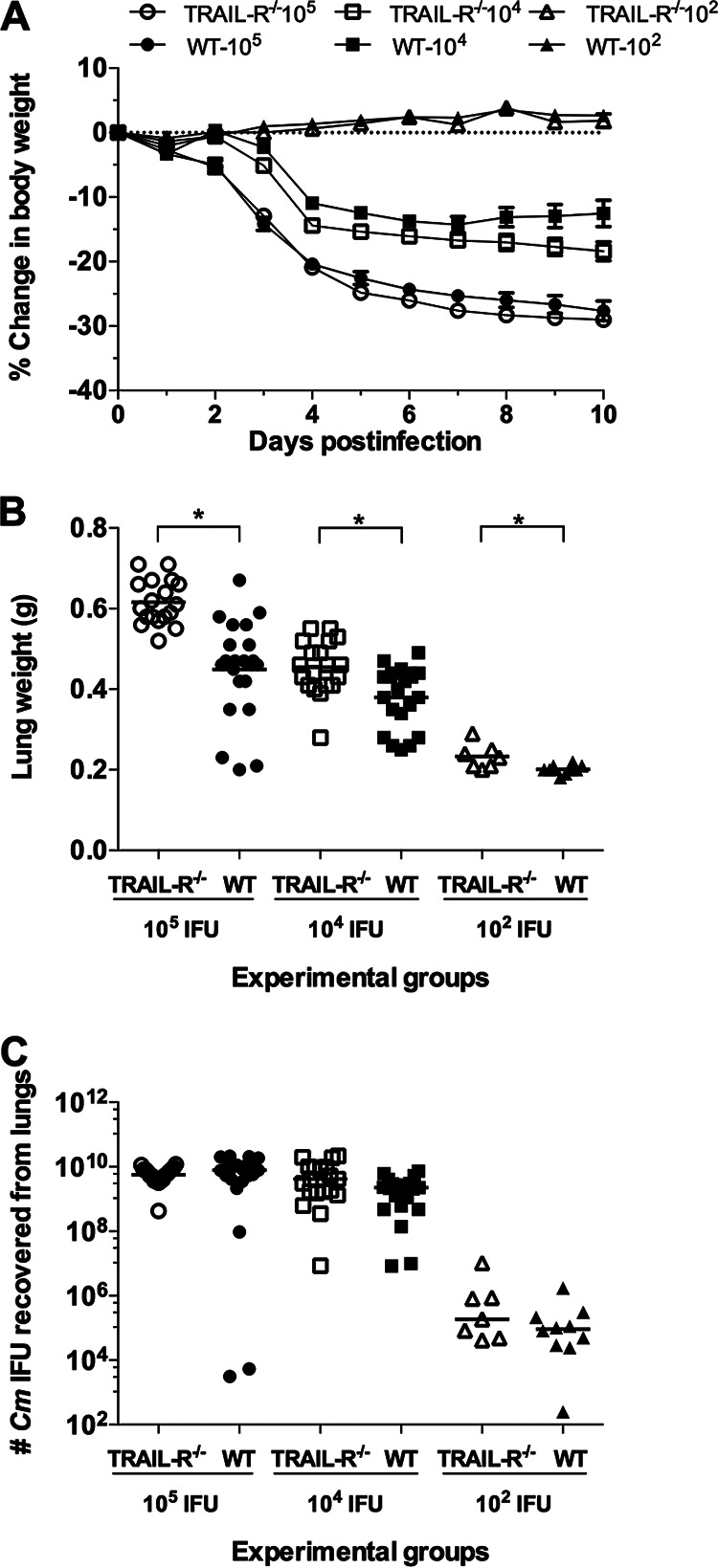
Changes in body and lung weight and number of C. muridarum IFU recovered from the lungs of WT and *TRAIL-R*^−/−^ mice following primary intranasal infection with 10^2^, 10^4^, or 10^5^ C. muridarum IFU. (A) Changes in body weight, determined daily for a total of 10 days. (B) Weight of the lungs at 10 dpi. The horizontal lines correspond to the mean number of grams. The symbols denote individual mice. *, *P* < 0.05 by the Student’s *t* test. (C) Number of C. muridarum IFU recovered from the lungs at 10 dpi. The horizontal lines represent the median number of C. muridarum IFU. The symbols denote individual mice.

As shown in Fig. 4A, all mice infected i.n. with 10^2^ C. muridarum IFU maintained their body weight during the 10 days of observation. Mice inoculated with 10^4^ or 10^5^ C. muridarum IFU quickly lost body weight for the first 4 days, and weight loss was then moderate until 10 dpi, when the animals were euthanized. No significant body weight differences were observed when comparing the WT and *TRAIL-R*^−/−^ mice throughout the 10 days of the experiment (*P* > 0.05). At 10 dpi, the WT mice inoculated intranasally with 10^4^ C. muridarum IFU had lost 13.3% of their initial body weight, while the *TRAIL-R*^−/−^ mice had lost 18.4% (*P* < 0.1).

As a parameter indicative of the local inflammatory responses, the weight of the lungs was determined following euthanasia at 10 dpi (Fig. 4B and Table S6). The lungs from the *TRAIL-R*^−/−^ mice were significantly heavier than those of the WT mice at the three different C. muridarum doses used, indicative of more robust inflammatory responses in the *TRAIL-R*^−/−^ mice. For instance, the median weight of the lungs of WT mice inoculated with 10^4^ C. muridarum IFU was 0.38 g, while for the *TRAIL-R*^−/−^ mice, it was 0.45 g (*P* < 0.05).

No significant differences in the median number of C. muridarum IFU recovered from the lungs at 10 dpi were observed between the two groups of mice (Table S6 and Fig. 4C). For example, the median number of IFU recovered from the WT mice infected i.n. with 10^4^ C. muridarum IFU was 2.25 × 10^9^ and from the *TRAIL-R*^−/−^ mice, 4.16 × 10^9^ (*P* < 0.1).

### Characterization of the disease burden following a secondary respiratory infection with C. muridarum.

To determine if the *TRAIL-R*^−/−^ mice mounted stronger or weaker protective immune responses than the WT animals, following a primary i.n. infection with 10^4^ IFU of C. muridarum, the mice were infected again i.n. 6 weeks later with the same number of IFU.

**FIG 5:**
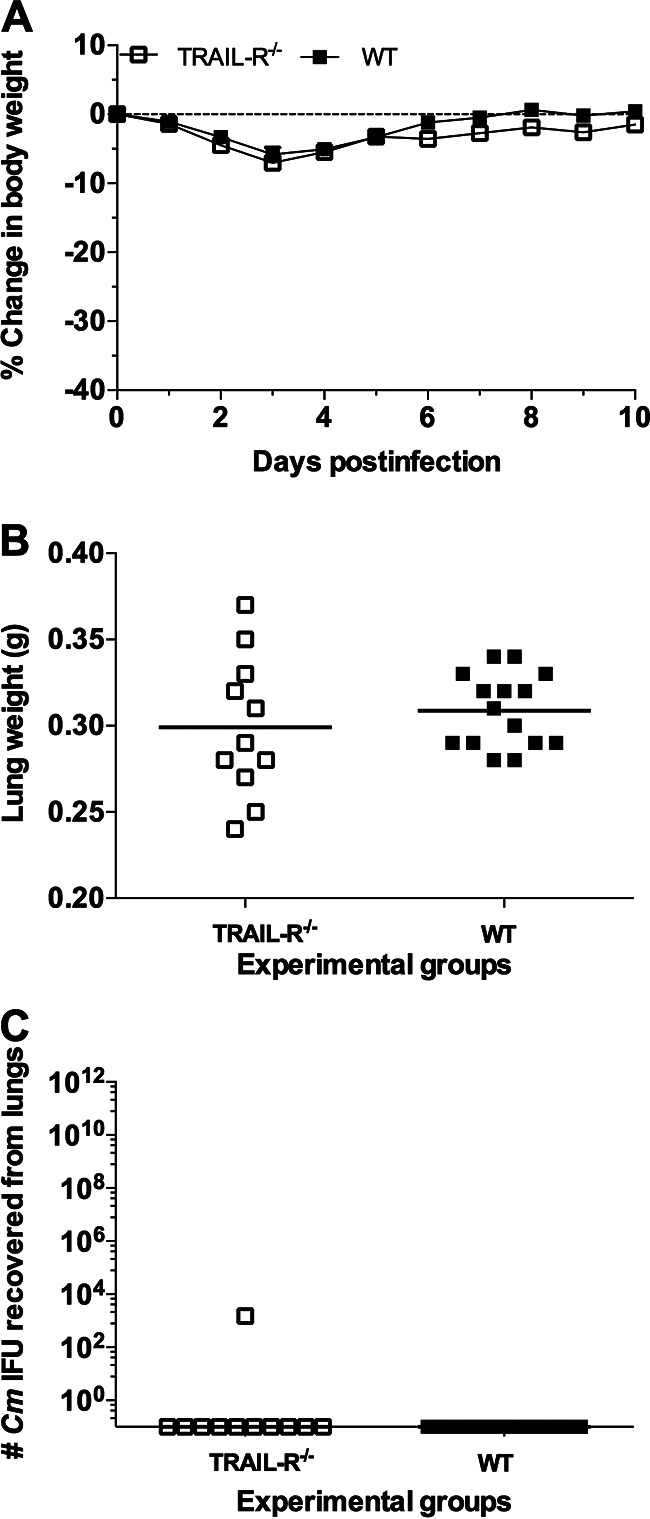
Changes in body and lung weight and number of C. muridarum IFU recovered from WT and *TRAIL-R*^−/−^ mice following primary and secondary intranasal infections with 10^4^ C. muridarum IFU. WT and *TRAIL-R*^−/−^ mice were inoculated in the nostrils with 10^4^ C. muridarum IFU and 6 weeks later were infected again in the nostrils with the same number of C. muridarum IFU. (A) Changes in body weight. (B) Mice were euthanized at 10 days following the secondary intranasal infection and their lungs collected and weighed. The horizontal lines correspond to the mean lung weight in grams. The symbols denote individual mice. (C) Number of C. muridarum IFU determined at 10 dpi. The horizontal lines represent the median number of C. muridarum IFU. The symbols denote individual mice.

As shown in Fig. 5A to C and Table S7, no significant differences in changes in body weight, lung weight, or number of IFU recovered from the lungs were observed between the WT and the *TRAIL-R*^−/−^ mice following a secondary infection. The percentage body weight loss in the WT mice at 10 dpi was 0.4%, while for the *TRAIL-R*^−/−^ mice, it was 1% (*P* > 0.05). The mean weight of the lungs of the WT and *TRAIL-R*^−/−^ mice at 10 dpi following a secondary infection was the same, 0.31 g (*P* > 0.05), and the median number of C. muridarum IFU was below the level of detection (<50 IFU/lung) for both groups of animals (*P* > 0.05).

## DISCUSSION

The goal of this study was to determine if WT and *TRAIL-R*^−/−^ C57BL/6 mice differ in their immune responses to primary and secondary vaginal and respiratory C. muridarum infections. Specifically, we were interested in determining their susceptibility to infection, ability to control the infection, and their adeptness at modulating long-term sequelae. For the most part, no significant differences were found between the two types of mice.

The presence of pathogen-associated molecular patterns (PAMPs), such as lipooligosaccharides (LOS), lipoproteins, and nucleic acids, in *Chlamydia* that bind to pattern recognition receptors (PRRs) in the host cells, like Toll-like receptors (TLRs) and Nod-like receptors (NLRs), promotes inflammatory responses (27–31). Once activated, PRRs interact with adaptors such as MyD88 that stimulate additional adaptor proteins, including the IL-1 receptor-associated kinases (IRAK) and TNF receptor-associated factor 6 (TRAF6). TRAF6 then activates additional proteins, leading to the phosphorylation and degradation of the inhibitor of κΒα (I-κΒα), resulting in the release of activated nuclear factor κΒ (NF-κΒ). NF-κΒ translocates to the nucleus and stimulates the expression of pro-inflammatory molecules, including IL1α, IL-6, IL-8, and IL-18, and the granulocyte macrophage colony-stimulating factor (GM-CSF) (32–34). These inflammatory responses, more than C. trachomatis infection itself, may lead to long-term sequelae (32). Thus, inflammatory responses need to be controlled to avoid tissue damage.

To maintain homeostasis, several mechanisms dampen inflammation (35, 36). For example, soluble TLR2 and TLR4 can compete with the agonists and decrease the activation of TLR-mediated signals. Also, cytosolic regulators can control the TLR signaling pathways at the level of MyD88, IRAK1, TRAF6, and phophoinositide-3-kinase (37, 38).

TRAIL is a transmembrane protein that belongs to the TNF family and plays a role in the regulation of innate and adaptive immune responses, controlling infections and tumor growth suppression (38, 39). In humans, apoptosis occurs when TRAIL binds to two death receptors, TRAIL-R1 (DR1) or TRAIL-R2 (DR5). TRAIL can also bind to two decoy receptors, TRAIL-R3 (DcR1) and TRAIL-R4 (DcR2), without inducing apoptosis and also to a soluble receptor named osteoprotegerin (OPG) (38, 40, 41). In mice, there is only one full-length receptor, TRAIL-R (mDR5), which is homologous to human DR4 and DR5, and two decoy receptors, mDcTRAILR1 and mDcTRAILR2 (38). Both TRAIL and TRAIL-R are constitutively expressed in multiple tissues, including cells of the immune system such as natural killer (NK) cells, activated T cells, dendritic cells, and monocytes/macrophages (38, 39).

Starkey et al. (42) infected neonatal WT and TRAIL-deficient BALB/c mice intranasally with C. muridarum and followed the animals for a period of 2 months. At 10 days postinfection, the WT and *TRAIL*^−/−^ animals had similar body weights, and the same numbers of C. muridarum IFU were recovered from their lungs. Histopathological analyses, at 15 days postinfection, showed a significantly higher inflammatory response in the lungs of the WT mice compared to the *TRAIL*^−/−^ animals. These results correlate with our observation that, at 10 days following a primary respiratory infection, the weight of the lungs of the *TRAIL-R*^−/−^ animals was significantly higher than that of the WT mice. Based on long-term follow-up of the mice, Starkey et al. (42) concluded that TRAIL increases inflammatory responses and mucus hypersecretion, which leads to alveolar enlargement and impaired lung function. In the genital tract model, however, we did not see differences in hydrosalpinx formation, following primary or secondary infections, between the WT and *TRAIL-R*^−/−^ mice. This could be due to the diverse levels of C. muridarum virulence in the respiratory tract versus the genital tract, since this organism is primarily a respiratory pathogen (43, 44). Alternatively, physiological differences in the immune response mounted in the respiratory versus the genital tracts could account for these findings.

In the genital model, the only statistically significant difference observed was when mice were inoculated with 1 × 10^2^ C. muridarum IFU. At this very low dose, the vaginal shedding in the *TRAIL-R*^−/−^ mice was significantly higher than that in the WT animals. However, when mice where inoculated with 2 × 10^2^ C. muridarum IFU, no significant differences were observed between the WT and *TRAIL-R*^−/−^ mice. Thus, although at a very low inoculum, there may be significant differences in susceptibility to infection with Chlamydia between these two strains of mice, we question whether this difference is biologically relevant.

In summary, human TRAIL-R1 correlated with infection in humans, whereas TRAIL-R did not show a significant effect in infected mice. In this sense, the difference between human TRAIL-R1 and murine TRAIL-R is reminiscent of the difference between human and murine Chlamydia strains with regard to the role played by indoleamine dioxygenase (IDO) in chlamydial infection. In human epithelial cells, gamma interferon (IFN-γ) induces IDO expression, which inhibits the growth of Chlamydia by depleting host tryptophan pools. Human Chlamydia strains, but not murine strains, avoid this response through the production of tryptophan synthase (45). Thus, both the host and pathogen species play critical roles in the outcome of the infection. We conclude that the results of TRAIL-R during chlamydial infection *in vitro* do not correlate with the effects of the receptor in mice. More studies are needed to determine whether human TRAIL receptors play a role in inflammation during chlamydial infection.

## MATERIALS AND METHODS

### Mice.

Breeding pairs of *TRAIL-R*^−/−^ mice, with a C57BL/6 background, were obtained from Astar Winoto (University of California, Berkeley) and were bred at the University of California, Irvine. WT C57BL/6 mice were purchased from Jackson Laboratory. All mouse experiments were approved by the University of California, Irvine Institutional Animal Care and Use Committee (UCI IACUC).

### Stocks of C. muridarum.

A stock of C. muridarum (strain Nigg II; previously called C. trachomatis mouse pneumonitis [MoPn] biovar) was purchased from the American Type Culture Collection (ATCC; Manassas, VA). C. muridarum was grown in HeLa 229 cells, and elementary bodies (EB) were purified using published procedures (43, 46). C. muridarum EB were stored at −80°C and titered in HeLa 229 cells (47).

### Genital primary and secondary infections with C. muridarum.

To determine the role that TRAIL-R may play in a genital primary infection, anesthetized groups of 8- to 10-week-old WT and *TRAIL-R*^−/−^ mice were inoculated intravaginally with 10^2^, 10^3^, 10^4^, or 10^5^ IFU of C. muridarum in 20 μL minimum essential medium (MEM) (47, 48). Following the genital inoculation, vaginal samples for culture were collected weekly with calcium alginate swabs for a period of 6 weeks, placed into 0.2 mL sugar phosphate glutamine (SPG) buffer, and frozen at −80°C. The specimens were cultured in 48-well plates seeded with HeLa 229 cells for 30 h, and C. muridarum IFU were fixed with methanol, stained with monoclonal antibody (MAb) MoPn-40, and counted as described (47). The limit of detection was two C. muridarum IFU/culture. Hydrosalpinx formation was established by visual inspection of the upper genital tract.

Adaptive humoral immune responses of WT and *TRAIL-R*^−/−^ mice were compared by first infecting the animals vaginally with 2 × 10^2^ IFU of C. muridarum and 6 weeks later, challenging them intravaginally with 10^3^ IFU of C. muridarum. Vaginal cultures were collected as above.

Before each vaginal infection, the estrus cycle was synchronized in diestrus by injecting each mouse subcutaneously with 2 mg Depo-Provera (medroxy-progesterone acetate) 4 days before each vaginal infection. All experiments were replicated.

### Determination of antibody responses following vaginal infections.

Blood was terminally collected from the heart at 6 weeks following primary and secondary vaginal infections, and C. muridarum-specific antibody titers in serum were determined using an enzyme-linked immunosorbent assay (ELISA) (47, 49). In brief, 96-well plates were coated with 100 μL of live C. muridarum EB in phosphate-buffered saline (PBS) at a concentration of 10 μg protein/mL. Serum (100 μL) was added to each well in 2-fold serial dilutions. After incubation at 37°C for 1 h, the serum was discarded and the wells washed three times with PBS. The plates were incubated with horseradish peroxidase-conjugated goat antimouse IgG, IgG1, or IgG2c antibodies (BD Pharmingen, San Diego, CA). The binding was measured in an EIA reader (Multiskan; LabSystems, Helsinki, Finland) using ABTS [2, 2′-azino-bis-(3-ethylbenzthiazoline-6-sulfonate)] (Sigma-Aldrich, St. Louis, MO) as the substrate. Preinfection serum samples were used as negative controls.

### Intranasal primary and secondary infections with C. muridarum.

To determine the median lethal dose (LD_50_) using the Reed-Muench method (50), groups of 10- to 12-week-old anesthetized male WT and *TRAIL-R*^−/−^ mice were inoculated in the nostrils with 10^4^, 10^5^, 10^6^, or 10^7^ of C. muridarum IFU. The animals were followed for 10 days and then euthanized.

To evaluate the effects of a primary respiratory infection, WT and *TRAIL-R^−/−^* mice were inoculated in the nostrils with 10^2^, 10^4^, or 10^5^ IFU. The systemic effects of the infection were assessed by determining the body weight changes daily for 10 days. Following euthanasia, to determine the local inflammatory responses, the weight of their lungs was measured. The bacterial burden was measured by homogenizing the lungs and infecting HeLa 229 monolayers in 10-fold serial dilutions. After 30 h at 37°C, the monolayers were stained with C. muridarum-specific MAb MoPn-40 and the number of IFU counted. The limit of detection was 50 C. muridarum IFU/mouse lung.

To determine if *TRAIL-R*^−/−^ mice mount adaptive immune responses equivalent to those of WT animals, 6 weeks following the primary infection with 10^4^ IFU, 12- to 14-week-old male mice were inoculated i.n. with 10^4^ C. muridarum IFU and followed as described above. The experiment was replicated.

### Statistical analyses.

The Mann-Whitney U test, Fisher’s exact test, repeated measure of ANOVA, and Student’s *t* test were used for statistical analysis using the program SigmaStat version 3.5.
