# Supplementary material for: Role of TRAIL-R in Primary and Secondary Genital and Respiratory Chlamydia muridarum Infections in Mice
Source: Microbiol Spectr. 2022 Jul 25;10(4):e01617-22. doi: 10.1128/spectrum.01617-22 (PMC9431660; doi:10.1128/spectrum.01617-22)
Supplement: Supplemental file 1 — Supplemental material. Download spectrum.01617-22-s0001.pdf, PDF file, 0.1 MB [file spectrum.01617-22-s0001.pdf]

Supplemental Table 1A: Vaginal shedding of WT and TRAIL-R$^{-/-}$ mice following a primary intravaginal infection with various doses of *C. muridarum* IFU.

Mouse type	# IFU	# mice	Median number of *C. muridarum* IFU collected post infection days (d) (range)								
			d4	d7	d11	d15	d18	d21	d28	d35	d42
TRAIL-R$^{-/-}$	1x10^5	20	106,215[a] (15,392-3,57,006)	176,540 (31,044-686,760)	381,096[b] (28,388-1,355,166)	182,368 (1,184-924,410)	6,882 (<2-466,570)	488 (<2-465,600)	<2[b] (<2-2,235)	<2 (<2-11,544)	<2 (<2-<2)
WT	1x10^5	26	237,606 (14,762-1,190,418)	204,771 (52,246-1,307,560)	1,038,780 (57,312-4,441,680)	155,545 (222-2,160,190)	8,577 (<2-372,480)	377 (<2-2,91,000)	<2 (<2-14)	<2 (<2-3,108)	<2 (<2-<2)
TRAIL-R$^{-/-}$	1x10^4	20	165,369[b] (<2-464,466)	73,431[b] (<2-407,154)	171,339[a] (<2-808,338)	59,103[b] (<2-776,100)	692 (<2-65,232)	355 (<2-8,140)	<2 (<2-866)	<2 (<2-2,738)	<2 (<2-<2)
WT	1x10^4	20	256,113 (<2-736,698)	161,190 (<2-592,224)	450,735 (<2-1,432,800)	32,163 (<2-146,862)	1,887 (<2-106,266)	152 (<2-117,012)	<2 (<2-148)	<2 (<2-<2)	<2 (<2-<2)
TRAIL-R$^{-/-}$	1x10^3	19	14,496 (<2-213,726)	82,386 (<2-705,654)	111,042 (<2-441,780)	59,700 (<2-512,226)	947 (<2-296,112)	614 (<2-139,698)	<2[a] (<2-151,638)	<2 (<2-6,364)	<2 (<2-37)
WT	1x10^3	20	48,357 (<2-943,260)	201,189 (<2-1,322,952)	223,278 (<2-1,307,430)	38,807 (<2-2,770,080)	3515 (<2-132,534)	344 (<2-146,862)	<2 (<2-437)	<2 (<2-10)	<2 (<2-<2)
TRAIL-R$^{-/-}$	1x10^2	22	<2[a] (<2-<2)	<2(<2-<2)	<2 (<2-<2)	<2[a] (<2-<2)	<2 (<2-<2)	<2 (<2-<2)	<2 (<2-<2)	<2 (<2-2)	<2 (<2-<2)
WT	1x10^2	22	<2 (<2-290,142)	<2 (<2-219,696)	<2 (<2-1,361,160)	<2 (<2-171,936)	<2 (<2-54,924)	<2 (<2-25972)	<2 (<2-6142)	<2 (<2-<2)	<2 (<2-<2)

[a] P<0.05 by the Mann-Whitney U test compared to the WT mice receiving the same number of *C. muridarum* IFU.
[b] P<0.1 by the Mann-Whitney U test compared to the WT mice receiving the same number of *C. muridarum* IFU.

Supplemental Table 1B: Vaginal culture and hydrosalpinx formation of WT and TRAIL-R$^{-/-}$ mice following a primary intravaginal infection with various doses of *C. muridarum* IFU.

Mouse type	#IFU/ mouse	Mice with positive cultures/total (%)	Median no. days to negative culture (range)	No. of positive cultures/total (%)	#IFU shed/mouse median (range)	# mice with hydrosalpinx (%)
TRAIL-R$^{-/-}$	$1x10^5$	20/20 (100)	35 (21-42)[a]	121/180 (67)	1,076,484 (308,126-2,666,330)[a]	15/20 (75)
WT	$1x10^5$	26/26 (100)	27 (18-42)	144/220 (65)	2,005,278 (331,864-5,904,896)	18/26 (69)
TRAIL-R$^{-/-}$	$1x10^4$	19/20 (95)	27 (21-42)[a]	120/180 (67)	541,581 (<2-2,008,018)[a]	18/20 (90)
WT	$1x10^4$	19/20 (95)	27 (4-35)	108/180 (60)	1,223,424 (<2-2,071,590)	14/20 (70)
TRAIL-R$^{-/-}$	$1x10^3$	15/19 (79)	27 (4-42)	101/171 (59)	478,024 (<2-2,014,416)[b]	8/19 (42)[d]
WT	$1x10^3$	16/20 (80)	27 (4-42)	104/180 (58)	957,606 (<2-2,963,142)	15/20 (75)
TRAIL-R$^{-/-}$	$1x10^2$	0/22 (0)	4 (4-42)	2/198 (1)[c]	<2 (<2-2)	0/22 (0)
WT	$1x10^2$	3/22 (14)	4 (4-35)	27/198 (14)	<2 (<2-1,522,930)	2/22 (9)

[a] P<0.05 by the Mann-Whitney U test compared to the WT mice receiving the same number of *C. muridarum* IFU.
[b] P<0.1 by the Mann-Whitney U test compared to the WT mice receiving the same number of *C. muridarum* IFU
[c] P<0.05 by the Fisher's Exact test compared to the WT mice receiving the same number of *C. muridarum* IFU.
[d] P<0.1 by the Fisher's Exact test compared to the WT mice receiving the same number of *C. muridarum* IFU.

Supplemental Table 2A: Vaginal shedding of WT and TRAIL-R$^{-/-}$ mice following a primary and a secondary intravaginal infection with *C. muridarum* IFU.

Mouse type	Infection	Median # *C. muridarum* IFU shed from vagina post infection days (range)								
		d4	d7	d11	d15	d18	d21	d28	d35	d42
TRAIL-R$^{-/-}$	Primary	<2 (<2-118,206)	<2 (<2-248,352)	<2 (<2-331,932)	<2 (<2-101,490)	<2 (<2-410,736)	<2 (<2-152,832)	<2 (<2-161,190)	<2 (<2-<2)	<2 (<2-<2)
WT	Primary	<2 (<2-643,566)	<2 (<2-199,398)	<2 (<2-442,974)	<2 (<2-463,272)	<2 (<2-319,992)	<2 (<2-403,572)	<2 (<2-2,220)	<2 (<2-<2)	<2 (<2-<2)
TRAIL-R$^{-/-}$	Secondary	<2[a] (<2-2,778)	<2[a] (<2-<2)	<2[a] (<2-<2)	<2[a] (<2-<2)	<2[a] (<2-<2)	<2[a] (<2-<2)	<2[a] (<2-<2)	<2 (<2-<2)	<2 (<2-<2)
WT	Secondary	<2[a] (<2-866)	<2[a] (<2-2,295)	<2[a] (<2-<2)	<2[a] (<2-<2)	<2[a] (<2-<2)	<2[a] (<2-<2)	<2[a] (<2-<2)	<2 (<2-<2)	<2 (<2-<2)

[a] P<0.05 by the Mann-Whitney U test compared to the primary infection of the same type of mice.

Supplemental Table 2B: Vaginal shedding and hydrosalpinx formation of WT and TRAIL-R$^{-/-}$ mice following a primary (2x10^2) and a secondary (10^3 IFU) intravaginal inoculation with *C. muridarum*.

Mouse type	Infection	Mice with positive cultures/total (%+)	Median no. days to negative culture (range)	No. of positive cultures/total (%+)	IFU shed/mouse median (range)	# mice with hydrosalpinx (%+)
TRAIL-R$^{-/-}$	Primary	13/20 (65)	25 (4-36)	56/180 (31.1)	12 (<2-759,687)	ND
WT	Primary	9/20 (45)	4 (4-36)	49/180 (27.2)	<2 (<2-1,792,544)	ND
TRAIL-R$^{-/-}$	Secondary	2/20 (10)[a]	4 (4-11)[b]	3/180 (1.7)[a]	<2 (<2-2,818)[b]	7/20 (35)
WT	Secondary	1/20 (5)[a]	4 (4-11)[b]	2/180(1.1)[a]	<2 (<2-3,161)[b]	5/20 (25)

ND= Not done as the same mice were reinfected vaginally.
[a] $P < 0.05$ by the Fisher's Exact test compared to the primary infection of the same type of mice.
[b] $P < 0.05$ by the Mann-Whitney U test compared to the primary infection of the same type of mice.

Supplemental Table 3: Serum antibody titers to *C. muridarum* EB 42 days following a primary vaginal infection.

Mouse strain	Inoculation dose (*Cm* IFU/mouse)	Geometric mean titer (range)			IgG2c/IgG1
		IgG	IgG1	IgG2c	
TRAIL-R$^{-/-}$	10^5	40,637 (25,600-51,200)	200 (100-800)	12,800 (6,400-25,600)	64
WT	10^5	32,254 (25,600-51,200)	400 (100-1,600)	8,063 (6,400-12,800)	20
TRAIL-R$^{-/-}$	10^4	51,200 (51,200-51,200)	159 (100-200)	12,800 (12,800-12,800)	81
WT	10^4	51,200 (25,600-102,400)	126 (100-200)	10,159 (3,200-25,600)	81
TRAIL-R$^{-/-}$	10^3	25,600 (25,600-25,600)	283 (100-800)	8,063 (6,400-12,800)	28
WT	10^3	40,637 (25,600-51,200)	141 (100-200)	12,800 (12,800-12,800)	91
TRAIL-R$^{-/-}$	10^2	112 (100-200)[a]	<100[a]	<100[a]	-
WT	10^2	3,592 (100-25,600)	238 (<100-400)	3,676 (200-12,800)	15
TRAIL-R$^{-/-}$	0	<100	<100	<100	-
WT	0	<100	<100	<100	-

[a] $P < 0.05$ by the Mann-Whitney U test compared to the WT mice.

Supplemental Table 4: Serum antibody titers to *C. muridarum* EB following primary and secondary vaginal infections.

Mouse strain	Inoculation dose (*Cm* IFU/mouse)	Days p.i.	Geometric mean titer (range)			IgG2c/IgG1
			IgG	IgG1	IgG2c	
TRAIL-R$^{-/-}$	1° infection 2x10^{2}	63	12,800 (12,800 -12,800)	800 (400-1,600)	18,102 (12,800-25,600)	23
WT	1° infection 2x10^{2}	63	25,600 (25,600-25,600)	800 (400-1,600)	12,800 (12,800-12,800)	16
TRAIL-R$^{-/-}$	2° infection 1x10^{3}	122	12,800 (12,800 -12,800)	1,600 (1,600-1,600)	25,600 (25,600-25,600)	16
WT	2° infection 1x10^{3}	122	36,204 (25,600-51,200)	800 (800-800)	18,102 (12,800-25,600)	23

Supplemental Table 5: Vaginal antibody titers to *C. muridarum* EB following primary and secondary vaginal infections.

Mouse strain	Inoculation dose (*Cm* IFU/mouse)	Days p.i.	Vaginal wash titer	
			IgG	IgA
TRAIL-$R^{-/-}$	1° infection $2x10^2$	63	160	160
WT	1° infection $2x10^2$	63	320	320
TRAIL-$R^{-/-}$	2° infection10^3	122	320	320
WT	2° infection10^3	122	160	320

Supplemental Table 6: Disease burden and yields of *C. muridarum* IFU following a primary i.n. infection of WT and TRAIL-R$^{-/-}$ mice.

Mouse strain	*Cm* #IFU	% Body weight change mean±SE	Lung weight (g) mean±SD	# of *C. muridarum* IFU in the lungs		
				Median	min	max
TRAIL-R$^{-/-}$	10^5	-29.0±0.6	0.62±0.06[a]	5,696,825,000	423,325,000	11,918,500,000
WT	10^5	-28.5±1.5	0.46±1.0	7,990,000,000	5369	21,165,000,000
TRAIL-R$^{-/-}$	10^4	-18.4±1.6[b]	0.45±0.07[a]	4,162,500,000[c]	8,510,000	21,624,500,000
WT	10^4	-13.3±2.0	0.38±0.08	2,250,850,000	8,325,000	7,310,100,000
TRAIL-R$^{-/-}$	10^2	1.8±1.0	0.23±0.03[a]	187,550	41,745	10,285,000
WT	10^2	2.7±0.7	0.20±0.01	91,355	250	1,712,150

[a] P<0.05 by the Student's *t* test compared to the WT mice receiving the same number of *C. muridarum* IFU.
[b] P<0.1 by the Student's *t* test compared to the WT mice receiving the same number of *C. muridarum* IFU.
[c] P<0.1 by the Mann-Whitney U test compared to the WT mice receiving the same number of *C. muridarum* IFU.

Supplemental Table 7: Disease burden and yields of *C. muridarum* IFU of WT and TRAIL-R$^{-/-}$ mice following a secondary (10^4 IFU) i.n. infection.

Mouse strain	Infection	% Body weight change Mean ± SE	Lung weight Mean ± SD	# of *C. muridarum* IFU recovered		
				Median	Min	Max
TRAIL-R$^{-/-}$	Secondary	-1.0±0.5	0.31±0.04	<50	<50	1,500
WT	Secondary	0.4±0.4	0.31±0.02	<50	<50	<50
